# Comparative functional analysis of macrophage phagocytosis in Dagu chickens and Wenchang chickens

**DOI:** 10.3389/fimmu.2023.1064461

**Published:** 2023-02-06

**Authors:** Jin Zhang, Qiao Wang, Qinghe Li, Zixuan Wang, Maiqing Zheng, Jie Wen, Guiping Zhao

**Affiliations:** Key Laboratory of Animal (Poultry) Genetics Breeding and Reproduction, Ministry of Agriculture and Rural Affairs, Institute of Animal Sciences, Chinese Academy of Agricultural Sciences, Beijing, China

**Keywords:** macrophage, phagocytosis, chicken, *Salmonnella*, transcriptomics, genomic selection

## Abstract

Phagocytosis of macrophages constitutes a powerful barrier to innate immunity. Differences in the phagocytic function of macrophages among chicken breeds have rarely been reported, and the molecular mechanisms underlying phagocytosis remain poorly understood. This study compared functional difference of macrophages in Dagu chickens, originated in Zhuanghe, Liaoning Province, China, and Wenchang chickens, originated from Hainan Island in the South China Sea, and explored the potential molecular mechanisms by integrated analysis of mRNA expression profiles of macrophages and whole genome sequencing. Immunological parameters in peripheral blood indicated that Dagu chickens were more resistant to *Salmonella* challenge at 28 days old. Phagocytosis index and phagocytosis rate of macrophages displayed Dagu chickens performed a significantly higher phagocytic ability of macrophages at 14 and 28 days old. Furthermore, comparative analysis of mRNA expression profiles of macrophages of two breeds at 28 days old revealed that 1136 differentially expressed genes (DEGs), and 22 DEGs (e.g., H2AFZ, SNRPA1, CUEDC2, S100A12) were found to be hub genes regulating phagocytosis by participating in different immunological biological signaling pathways. In addition, many DEGs and hub genes were under strong differentiation in genome between two breeds, the H2AFZ gene was an intersection of DEGs and hub genes. These results provided a comprehensive functional comparison and transcriptomic profiles of macrophages in Chinese native chicken breeds, and deepened our understanding of the genetic mechanism of innate immunity.

## Introduction

Macrophages play a crucial role in innate and acquired immunity, especially against microbial infections (1–3). Macrophages are major members of mononuclear phagocytic system lineage, and phagocytosis is the classical function for this cell type. Macrophages perform phagocytic functions *via* specific receptors present on their surfaces, which are capable of binding specific targets for phagocytosis. Macrophages recognize and bind bacteria through pattern recognition receptor (PRR) interaction with pathogen-associated molecular patterns (PAMP) (4). Macrophages also have other specific receptors, such as mannose receptors, scavenger receptors and Fc receptors (5). To kill and degrade bacteria, once bound to bacteria, macrophages swallow them in the form of phagosomes into cytoplasm following phagocytosis. Macrophages trigger intracellular signaling through cytokines and activate the processing pathway for antigen presentation in macrophages, which in turn switches on acquired immune responses (6–8).

Chicken macrophage function is regulated by the genetic composition of chicken. There have been studies on the differences in macrophage function between different chicken lines. For example, Qureshi, et al. reported that macrophages from chicken lines with different B-complex alleles performed different chemotactic, phagocytic, and bactericidal responses; furthermore, the production of macrophage tumor necrosis factor was also affected by genotype (9, 10). The expression and activity of inducible nitric oxide synthase (iNOS) were associated with Toll-like receptor-4 expression of macrophages against various bacteria in different chicken genetic lines (11). Chicken major histocompatibility complex (MHC) affected certain properties of chicken macrophage function in several white Leghorn chicken genetic lines (9). Macrophages expressed more rapid and greater proinflammatory chemokines and cytokines in inbred chickens that are resistant to salmonellosis (12). However, the differences of macrophages function and genetic mechanism in different chicken breeds remain poorly reported.

Chicken breeds perform genetic adaptations to their local environmental conditions after a long period of selection pressure (13, 14). Study reported that tropical climates was an important driver not only for shaping the chicken thermal adaptation but also immune adaptation to the defense against zoonotic diseases in chickens (15). China has rich aboriginal chicken breeds, and these breeds possess diverse traits adapted for local environments. Dagu chickens, originated in Zhuanghe, Liaoning Province, China, are currently mainly raised in parts of northeast China, Hebei, and Inner Mongolia (16). Dagu chickens are famous as the king of the northeast chickens and have significant advantages, such as adaptability to rough forage, cold resistance and high disease resistance. Wenchang chickens, originated from Hainan Island in the South China Sea, are known for excellent meat and rich flavor (17). However, there are few reports on the disease resistance of Wenchang chickens. Long-term north-south and human-geographical differences are the driving forces behind the formation of the two breeds. Thus, the two native chicken breeds can offer a suitable model to study the difference in disease resistance and the relationship between macrophage function and genetic factors.

To explore the difference in the immune response and macrophage function between Dagu chickens and Wenchang chickens and reveal the relevant genetic mechanism, we detected the immunological parameters and phagocytic function of macrophages against *Salmonella* infection, integrated transcriptome of macrophages and genome to uncover the immune characteristics and potential genetic basis of macrophage phagocytosis. The study was conducive to the development of local genetic resource characteristics and the breeding of disease-resistant chicken breeds.

## Materilas and methods

### Animal and experimental design

A total of 200 chickens were obtained from the Poultry Institute of the Chinese Academy of Agricultural Sciences (Yangzhou, China), including 100 Dagu chickens and 100 Wenchang chickens. The two chicken breeds were both from gene pool preservation populations. After hatching, all chicks were housed in an environmentally controlled room until 7 days of age and then transferred to sterilized isolation ventilated cages (IVCs) (IPQ-type 3 negative pressure isolator). The chicks were randomly divided into two groups: noninfected (25 individuals) and *Salmonella typhimurium* (ST)-infected (75 individuals) in each breed. These birds of the ST-infected group were randomly selected for *Salmonella* infection at 14 and 28 days of age. After *Salmonella* infection, the birds of each group were assigned to one IVC. The chicks received *ad libitum* access to feed and water throughout the experiment.

### Bacterial strain and infection

*Salmonella typhimurium* 21484 (China Industrial Microbial Culture Preservation Center, Beijing, China) was as infectious strain in this experiment. Before the infection assay, 100 µl cryopreserved bacterial solution was thawed at room temperature and then inoculated into 1000 ml Luria Bertani broth at 37°C with agitation (150 rpm) and cultured for 12 h. Then, 100 µl cultured bacterial liquid was inoculated into 1000 ml Luria Bertani broth and cultivated under the same conditions until the bacterial solution was recovered three times to achieve the highest activity. After centrifugation, the bacterial concentrate was resuspended using sterile phosphate-buffered saline (PBS). The colony-forming units (CFU) of *Salmonella* was determined by plating serial dilutions in triplicate on agar (37°C, overnight). In infection assay, the chicks from the ST-infected group were infected with 1.5×10^13^ CFU of ST/ml of PBS orally at 14 days old and 28 days old. The chicks from the noninfected group were given same volume of sterile PBS.

### White blood cell count and determination of the H/L ratio

The peripheral blood white blood cell count method was performed as described previously (18). Briefly, fresh blood was collected from the wing vein and 10 µl of which smeared on microscopic glass slides for each bird. Blood smears were air-dried and then stained with Wright’s Giemsa solution (G1020, Solarbio, Beijing) according to the manufacturer’s instructions. According to schematic diagram, two hundred leukocytes (heterophils, lymphocytes, and monocytes) were counted using a Leica DM500 microscope with a magnification of 1000× immersion oil. The percentage of monocytes was equal to the number of macrophages to the total number of leukocytes (heterophils, lymphocytes, and monocytes). H/L was the ratio of heterophils to lymphocytes.

### Detection of IL-17, IL-23, and G-CSF blood serum concentrations

The concentrations of IL-17, IL-23 and G-CSF in the serum were measured using enzyme- linked immunosorbent assay (ELISA) kits according to the manufacturer’s instructions (Bioswamp). The assay was performed in duplicate and included ten to eleven non-*Salmonella* infected and *Salmonella* infected chickens from Dagu chickens and Wenchang chickens at 24 hours after infection of 28 days old. In brief, optical density (OD) was measured by a microplate reader, standard curve was generated by dilute standard, the concentrations of IL-17, IL-23, and G-CSF in the serum were calculated according to the standard curves.

### Extraction and culture of primary chicken macrophages

The primary monocyte-derived macrophages of Wenchang chickens and Dagu chickens were extracted and cultured according to previous studies (19–21). Briefly, peripheral blood mononuclear cells (PBMCs) were isolated from fresh blood using chicken peripheral blood lymphocyte isolation kit (P8740, Beijing Solarbio Science & Technology Co., Ltd.) . After 6~8 h of static incubation in petri dish, adherent cells were washed twice with PBS to remove thrombocytes, nonadherent lymphocytes and other semi adherent cells. These adherent cells were primarily chicken monocytes. Subsequently, fresh Roswell Park Memorial Institute (RPMI)-1640 medium with 10% fetal bovine serum (FBS, Gibco), 5% chicken serum, 1% penicillin and streptomycin were added to the remaining monocytes. Chicken monocytes were then cultured in 12-well cell culture plates to generate macrophage differentiation for subsequent experiments. Treatment of all individuals during the experiment was carried out under consistent conditions.

### Phagocytosis assay

Assessment of phagocytic activity in macrophages was performed using a flow cytometry-based method. Briefly, 0.5 ml RPMI-1640 (containing 10% fetal bovine serum, 5% chicken serum, 1% penicillin and streptomycin) was added to each well covering macrophages in cell culture plates. Macrophages were stimulated with LPS (500 ng/ml) for 6 h, and then 4 µl of latex beads (Sigma-Aldrich, cat. L3030-1ML, 2.0 µm mean particle size; St. Louis, MO, USA) were mixed, and the samples were incubated for 2 h at 37°C. After incubation, the supernatant was removed (including nonadherent cells and excess fluorescent microspheres), and the cells were washed twice with cold PBS to stop phagocytosis. Next, adherent cells were trypsinized and transferred from the 12-well plates to test tubes containing 0.5 ml PBS. The samples were filtered through a 100-μm filter and analyzed by flow cytometry.

### Flow cytometry

The cell populations were distinguished based on forward scatter (FSC), side scatter characteristics (SSC) and GFP-positive cells. First, we set up the FSC-SSC 2D scatter plot, the macrophage gate was used to define the analyzed macrophage population by adjusting the voltage values of FSC and SSC, and the interference of other nucleated cells and cell debris was excluded to the maximum extent (**Supplementary Figure S1**). The fluorescence intensity of macrophages was detected by the GFP pathway, the fluorescence pathway of light emitted by fluorescent microspheres, obtaining 10000 macrophages per sample. Data are displayed in two-dimensional scatter plots and histograms. In the two-dimensional scatter diagram, the macrophage population without phagocytosis of fluorescent microspheres and the macrophage population phagocytosis of fluorescent microspheres could be delineated by setting gates. In the histogram, the macrophage population that did not phagocytose fluorescent microspheres and the macrophage population that phagocytosed fluorescent microspheres could be calibrated by using a scale, and the proportion of macrophages that did not phagocytose fluorescent microspheres and macrophages that phagocytosed different numbers of fluorescent microspheres was analyzed. The phagocytosis percentage and phagocytosis index were calculated according to the following formula:

(1) Percentage of phagocytosis (%) = number of macrophage phagocytic fluorescent microspheres/number of macrophages × 100%.
(2) Phagocytosis index = total number of phagocytosed fluorescent microspheres/total number of macrophages, the total number of phagocytic fluorescent microspheres = the total number of macrophages phagocytosed with 1 fluorescent microsphere × 1 + the total number of macrophages phagocytosed with 2 fluorescent microspheres × 2 + the total number of macrophages phagocytosed with 3 fluorescent microspheres × 3 + the total number of macrophages phagocytosed with 4 fluorescent microspheres × 5 + phagocytic 6 The total number of macrophages per fluorescent microsphere was × 6.

### RNA isolation and mRNA sequencing

To characterize the expression differences of related genes during phagocytosis, a total of 16 macrophage samples were collected from Wenchang chickens and Dagu chickens (n=8 each breed). Macrophages of non-infection group were subjected to RNA-seq during phagocytosis testing at 28 days old. Total RNA was extracted using TRIzol^®^ reagent (Invitrogen, Carlsbad, CA, USA). Using a NanoDrop 2000 (Thermo Fisher Scientific, Wilmington, DE, USA) to measure RNA concentration and purity. Using RNA Nano 6000 Assay Kit of the Agilent Bioanalyzer 2100 system (Agilent Technologies, Santa Clara, CA, USA) to assessed RNA integrity.

1 μg RNA per sample was used as input material. Using poly-T oligo-attached magnetic beads to purify mRNA from total RNA. Sequencing libraries were constructed using the NEBNext UltraTM RNA Library Prep Kit for Illumina (New England Biolabs, Ipswich, MA, USA). Finally, polymerase chain reaction (PCR) products were purified (AMPure XP system). Agilent Bioanalyzer 2100 system (Agilent, Santa Clara, CA, USA) was used to assessed the library quality. Library preparations were sequenced on an Illumina platform, and paired-end reads were generated. Approximately 6.35 Gigabases of clean data were obtained for each sample.

### Quality control and data analysis

In this step, clean data (clean reads) were obtained by removing reads containing adapters, reads containing poly-N sequences and low-quality reads from the raw data. The Q20, Q30, GC content and sequence duplication level of the clean data were calculated. All downstream analyses were based on high quality clean data. The clean data were further analysis with a bioinformatic pipeline tool, BMKCloud (www.biocloud.net).

### Differential expression analysis

Quantification of gene expression was estimated by fragments per kilobase of transcript per million fragments mapped (FPKM). EBSeq performed differential expression analysis between two groups. False discovery rate (FDR) < 0.05 and fold change ≥1.5 were set as the thresholds for significantly differentially expressed genes (DEGs).

### KEGG pathway analysis

Kyoto Encyclopedia of Genes and Genomes (KEGG) pathway enrichment analysis was performed by KOBAS 3.0 (22) to explore the function of differentially expressed genes (DEGs) and hub genes. *P* < 0.05 was the threshold for significant enrichment.

### Gene co-expression network analysis

All 16 transcriptomes data of macrophages were used to perform weighted gene co-expression network analysis. FPKM was utilized to construct a gene expression matrix (FPKM ≥ 1, variation of FPKM: cv ≥ 0.5). Weighted correlation network analysis (WGCNA) in the R package with the ‘unsigned’ weighted network type was carried out (23). Soft thresholding power option was set to 15, topology fit index R^2^ was first greater than 0.8, minimum module size was limited to 30. A minimum height of 0.22 was chosen to merge highly co-expressed modules. *P* < 0.05 was defined as the significance threshold. Module eigengenes (MEs) were referred to as ‘‘hub genes”, dependent on high intramodular connectivity values (|kME|≥0.8).

### Stimulation of HD11 cells with lipopolysaccharide

The HD11 cells were maintained in RPMI-1640 medium supplemented with 10% FBS, 5% chicken serum, 1% penicillin, 0.1% 2-Mercaptoethanol, 1% nonessential amino acid and 0.24% HEPES. The homogenized cell suspensions were seeded into 24-well plates at 5 × 10^5^ cells/well for a 24-well plate and maintained in humidified incubator at 37°C with 5% CO_2_. Cells were stimulated with lipopolysaccharide (LPS) at 100ng/ml for 6 hours and 12 hours.

### Quantitative real-time PCR

The total RNA of cells was extracted by Trizol reagent. RNA (1500ng) was reverse transcribed by cDNA synthesis kit (TIANGEN) for quantitative real time PCR. Primers were designed according to chicken coding region sequences and synthesized by The Beijing Genomics Institute (BGI), which are shown in **Table 1**. *ACTB* was selected as house-keeping gene. Quantitative real-time PCR was performed in triplicate using the Invitrogen PowerUp™ SYBR^®^ Green Master Mix (ABI). with the following cycle profile: 95 °C for 3 min, 40 cycles of 95 °C for 3 s, annealing temperature for 34 s in the QuantStuio 7 Flex Real-Time PCR System (Waltham, MA, USA). The results were analyzed by 2^−ΔΔCt^ method (24).

**Table 1:** Primers for qPCR.

Gene	Primer Sequence	Product Size (bp)
*ACTB*	F: 5’-GAGAAATTGTGCGTGACATCA-3’	152
	R: 5’-CCTGAACCTCTCATTGCCA-3’	
*CUEDC2*	F: 5’-GAAAGGCTTGGTGAAGCACG-3’	190
	R: 5’-CGACACCACCCTTCAAGTCA-3’	
*ADH4*	F: 5’-AAAGGCTGGGGAGTCAGTGT-3’	131
	R: 5’CTGTCTACGCTCTTCCAGCC-3’	
*S100A12*	F: 5’-TTGGTGAAGTGATGCTCCTGA-3’	114
	R: 5’-GGTTGTGCTGATGTTGGTGT-3’	
*AKR1E2*	F: 5’-GGAAGGCGTTGTGAAACGAG-3’	190
	R: 5’-TTCATCTGTCGGAAACAGGACA-3’	
*H2AFZ*	F: 5’-AAAAGCGGTGTCCCGTTCTC-3’	158
	R: 5’-CGAGAACCTCAGCTGTCAAGT-3’	
*IL1R2*	F: 5’-TCAAGACACTTTCCCTCGCTC-3’	135
	R: 5’-AAAGCCATGCCCTTACCTGT-3’	
*CX3CR1*	F: 5’-ATGCACCTGACCGAAACCAT-3’	123
	R: 5’-CAGGCATGGGCAGTACTTGA-3’	
*CCR5*	F: 5’-TACCTACGGCATCCTCACCA-3’	162
	R: 5’-AAGGCCAGGAATTGCTTCCA-3’	
*CENPF*	F: 5’-TCACAGTTGACACGCATGGA-3’	125
	R: 5’-TCCGGAAAGGTTCCATCATCA-3’	
*DIAPH3*	F: 5’-GCTCCAACTGTGTTTGGACAG-3’	151
	R: 5’-CGACAGCGGTTTTGGAACAA-3’	
*IQGAP3*	F: 5’-TGGCTTACGGTCTCTTGTGC-3’	174
	R: 5’-GCTACATTCTGGCGTCGTCT-3’	
*IL1β*	F: 5’-GCCTGCAGAAGAAGCCTCG-3’	172
	R: 5’-CTCAGGTCGCTGTCAGCAAAG-3’	

F, forward; R, reverse.

### Population differentiation evaluation and selective sweep analysis

DNA was extracted from the blood samples of 30 Dagu chickens and 30 Wenchang chickens by the conventional phenol-chloroform method. Sequenced on the Illumina HiSeq2500 platform after quality checking and quantification. The Genome Analysis Toolkit (GATK) (25) was employed for preprocessing and single nucleotide polymorphism (SNP) calling. Phylogenetic tree was built based on the weighted neighbor-joining method (26) and visualized using MEGA7.0 software (27) and iTOL software (28). SNPs were pruned based on linkage disequilibrium utilizing PLINK software (Version: 1.90) (29) with the options ‘–indep-pairwise 25 10 0.2 -mind 0.1 –maf 0.01’. Principal component analysis (PCA) was performed using PLINK software. Based on allele frequencies at variable sites, the average fixation index (FST) and nucleotide diversity(pi) were calculated to identify selection regions (–windows–40 kb –step 10 kb). For each window, the average FST values (30)and pi value (31) were assessed between the Dagu chickens and the Wenchang chickens. pi value was log_2_-transformed. The windows with the top 5% FST values and log_2_(pi-ratio) values were simultaneously shown as candidate outliers.

### Statistical analysis

Student’s *t*-*test* analyzed the significance of differences among groups. *P* < 0.05 (*), *P* < 0.01 (**) or *P* < 0.001 (***) implied a statistically significant difference respectively. Data were visualized using GraphPad Prism version 8 (GraphPad Software, San Diego, CA, USA) and R version 4.0.5 and expressed as mean ± standard deviation (SD) unless otherwise indicated.

## Results

### Comparison of immunological parameters between Dagu chickens and Wenchang chickens

**Figure 1:**
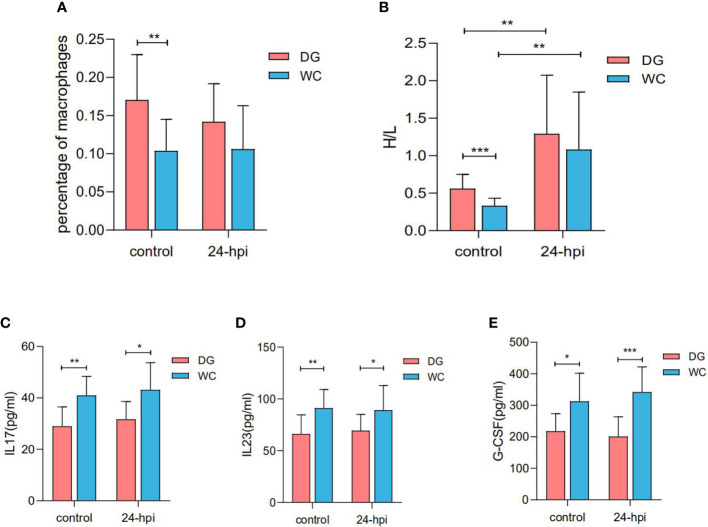
Immunological parameter differences between Dagu chickens and Wenchang chickens with or without *Salmonella* infection at 28 days old. **(A)** The percent of macrophages in peripheral blood differences between Dagu chickens and Wenchang chickens (n=12~13 per group); **(B)** Comparation of H/Lratio in peripheral blood between Dagu chickens and Wenchang chickens (n=12~13 per group); **(C)** Concentration differences of IL17 in serum between Dagu chickens and Wenchang chickens (n=10~11 per group); **(D)** Concentration differences of IL23 in serum between Dagu chickens and Wenchang chickens (n=10~11 per group); **(E)** Concentration differences of G-CSF in serum between Dagu chickens and Wenchang chickens (n=10~11 per group). P < 0.05 (*), P < 0.01 (**) or P < 0.001 (***).

To assess the level of immune response to infection, 28-day-old Dagu chickens and Wenchang chickens were challenged with *Salmonella*. Dagu chickens were characterized by a significantly increased percentage of macrophages and heterophils and lymphocytes (H/L) ratio in peripheral blood compared to Wenchang chickens. Additionally, the percentage of macrophages remained largely unchanged, while the H/L ratio increased significantly after *Salmonella* infection, which was consistent with previous reports (32) (**Figures 1A, B**). Furthermore, we compared interleukin 17 (IL17), interleukin 23 (IL23) and granulocyte colony-stimulating factor (G-CSF) concentrations in serum between Dagu chickens and Wenchang chickens. Unlike the above two indicators, the concentrations of IL-17, IL-23, and G-CSF were significantly higher in Wenchang chickens than in Dagu chickens before and after *Salmonella* infection (**Figures 1C–E**).

### Advantage of the stronger phagocytic ability of macrophages in Dagu chickens

**Figure 2:**
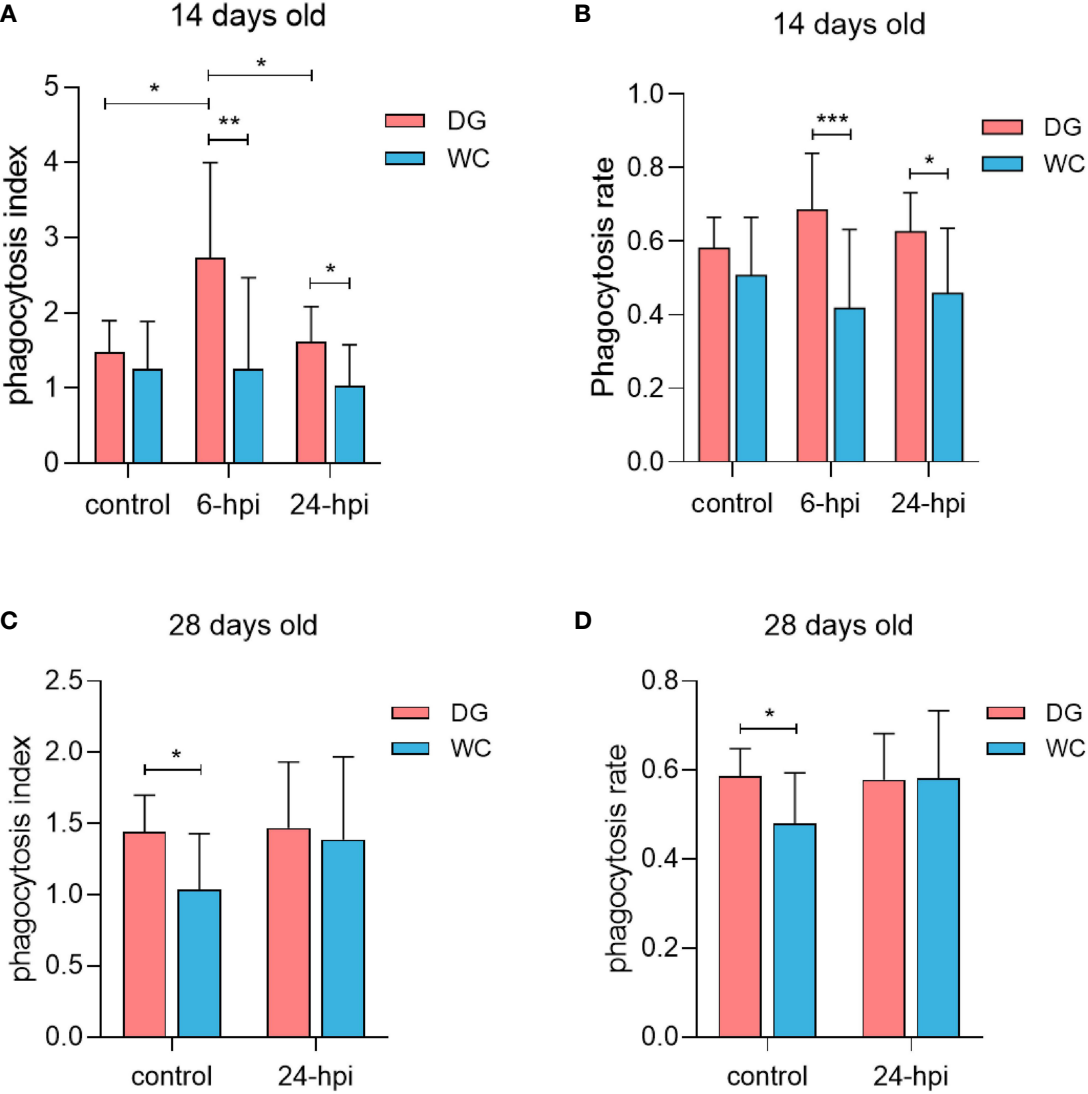
Stronger phagocytic ability of macrophages in Dagu chickens. Detection of phagocytic ability after isolation of peripheral blood macrophages in two breeds of chickens of different ages with or without *Salmonella* infection. **(A)** Comparison of phagocytosis index differences at 14 days old (n=8~14 per group); **(B)** Comparison of phagocytosis rate differences at 14 days old (n=8~14 per group); **(C)** Comparison of phagocytosis index differences at 28 days old (n=10~12 per group); **(D)** Comparison of phagocytosis rate differences at 28 days old (n=10~12 per group). P < 0.05 (*), P < 0.01 (**) or P < 0.001 (***).

To compare the phagocytic ability of peripheral blood macrophages between Dagu chickens and Wenchang chickens, 100 chicks of each breed were randomly divided into a control group and an infection group. At 14 days of age, the phagocytosis index of macrophages was significantly increased after *Salmonella* challenge, especially at 6 hours after infection (6 hpi) (**Figure 2A**). A similar trend existed for macrophage phagocytosis rate (**Figure 2B**). Remarkably, whether it was the phagocytosis index or phagocytosis rate of macrophages, Dagu chickens were always higher than Wenchang chickens, which was more significant after *Salmonella* challenge. Furthermore, at 28 days of age, under non-infection conditions, the phagocytosis index and phagocytosis rate of macrophages in Dagu chickens were significantly increased than Wenchang chickens, while the difference decreased after *Salmonella* challenge (**Figures 2C, D**). The results showed that the difference in the phagocytic ability of macrophages between Wenchang chickens and Dagu chickens at the early stage depended mainly on the stimulation of the external pathogenic environment. However, with age, differences in phagocytic ability were directly manifested and related to species specificity. Furthermore, the body weight of Dagu chickens was significantly increased compared to the body weight of Wenchang chickens at 14 days old, while the difference disappeared at 28 days old (**Supplementary Figure S2**). Overall, the advantage of the macrophage phagocytic ability of Dagu chickens was more prominent.

### mRNA sequencing profiling revealed differentially expressed genes associated with phagocytosis of macrophages

**Figure 3:**
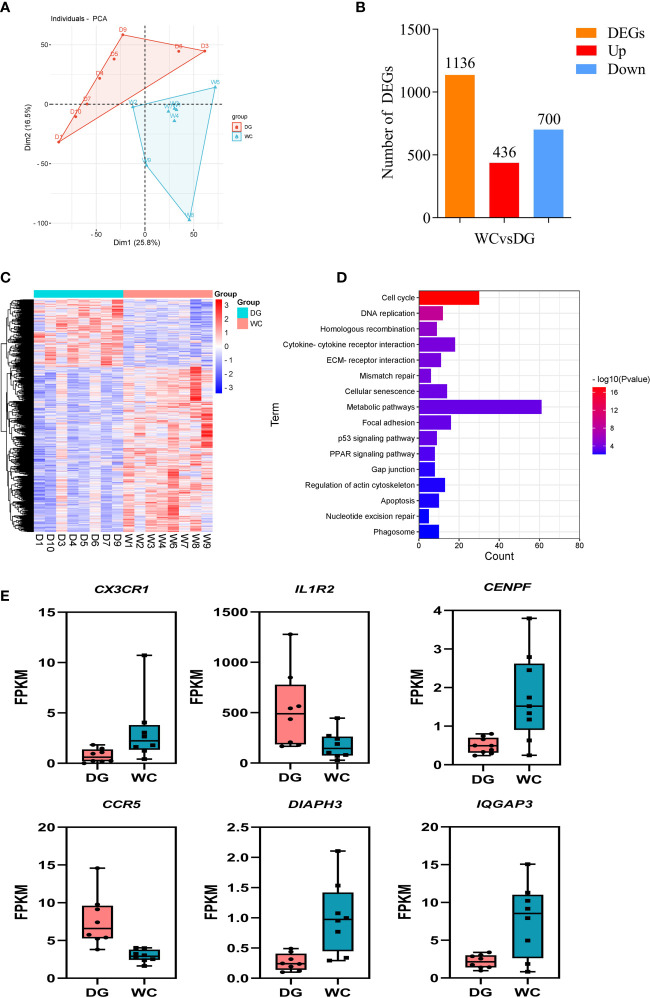
Differential expression analysis of macrophages in peripheral blood between Dagu chickens and Wenchang chickens. **(A)** Individual-PCA plot of all samples (Dagu chickens n=8; Wenchang chickens n=8). **(B)** Statistics of differentially expressed genes (DEGs). **(C)** Heatmap dendrogram based on the FPKM expression values of DEGs. Rows represent DEGs, while columns show samples. **(D)** Significant KEGG pathways of functional enrichment analysis among DEGs. **(E)** FPKM values of six representative DEGs in macrophages of Dagu chickens and Wenchang chickens.

Phagocytosis is an important cellular process that induces antimicrobial responses and modulates adaptive immunity (33). This process is coordinated by multiple gene signaling pathways to activate the internalization machinery (34). Analysis of the transcriptional diversity associated with phagocytosis to reveal the genetic mechanism of the difference in phagocytosis between Dagu chickens and Wenchang chickens should be possible. Based on this premise, we sought to integrated mRNA sequencing data to explore the functional factors involved in phagocytosis from peripheral blood macrophages of 8 Dagu chickens and 8 Wenchang chickens during phagocytosis. PCA showed that the two groups of individuals were distinctly clustered together (**Figure 3A**). 1136 differentially expressed genes (DEGs) were identified between the Dagu chickens and Wenchang chickens, of which Dagu chickens performed 436 upregulated genes and 700 downregulated genes compared to Wenchang chickens (**Figure 3B** **;** **Supplementary Table S1**). Hierarchical clustering was consistent with PCA based on all DEGs and showed that there were more downregulated than upregulated genes (**Figure 3C**). Functional enrichment analysis of the DEGs showed that many biological processes were related to phagocytosis, including the cell cycle, cytokine-cytokine receptor interaction, ECM-receptor interaction, apoptosis, and phagosome (**Figure 3D** **;** **Supplementary Table S2**).

Some interesting DEGs (expression fold changes greater than 2 between Dagu chickens and Wenchang chickens) were associated with immune responses, including *CX3CR1*, *IL1R2*, *CENPF*, *CCR5*, *DIAPH3*, and *IQGAP3* (**Figure 3E**). Analysis of above genes expression patterns revealed that multiple genes were likely involved in chicken macrophage biological functions in immunity.

### Co-expression modules delineate biological processes relevant to phagocytosis

**Figure 4:**
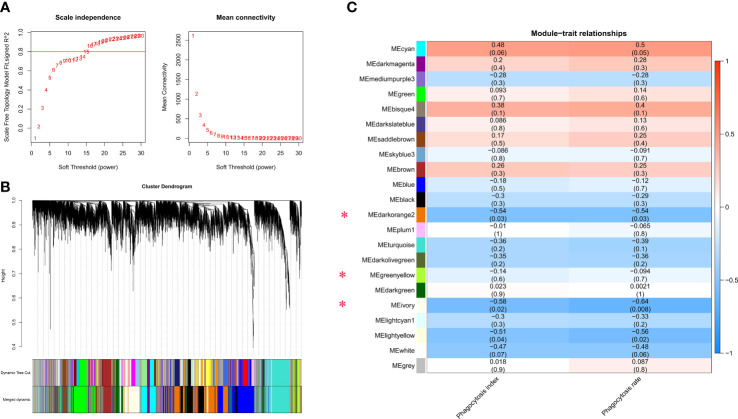
Gene coexpression module analysis of the macrophage phagocytosis index and phagocytosis rate. **(A)** Scale-free topology model and mean connectivity. **(B)** Cluster dendrogram revealed the module colors and the merged dynamics. **(C)** Module relationships with the phagocytosis index and phagocytosis rate. Modules significantly correlated with the phagocytosis index and phagocytosis rate are marked with a red asterisk. There are two values in each cell: the upper represents the absolute value of the correlation coefficient, and the lower represents the *P* value. Red and blue colors represent positive and negative correlations, respectively.

Weighted gene co-expression network analysis (WGCNA) was constructed to further elucidate biological pathways involved in phagocytosis. First, the best soft threshold of 15 was determined from the scale-free topological model and mean connectivity (**Figure 4A**). Then, using hierarchical clustering of genes based on the topological overlap matrix (TOM), a cluster dendrogram of co-expression network modules was generated (**Figure 4B**). A total of 22 co-expression modules were captured, three of which (MEdarkorange2, MEivory, and MElightyellow) were significantly correlated with the phagocytosis index, and four of which (MEcyan, MEdarkorange2, MEivory, and MElightyellow) were significantly correlated with the phagocytosis rate (Pearson correlation, *P* < 0.05). Notably, the phagocytosis index was strongly positively correlated with the phagocytosis rate (R^2^ = 0.77) (**Supplementary Figure S3**). The MEdarkorange2, MEivory, and MElightyellow modules were jointly identified modules, that were significantly negatively correlated with the phagocytosis index and phagocytosis rate (**Figure 4C**).

**Figure 5:**
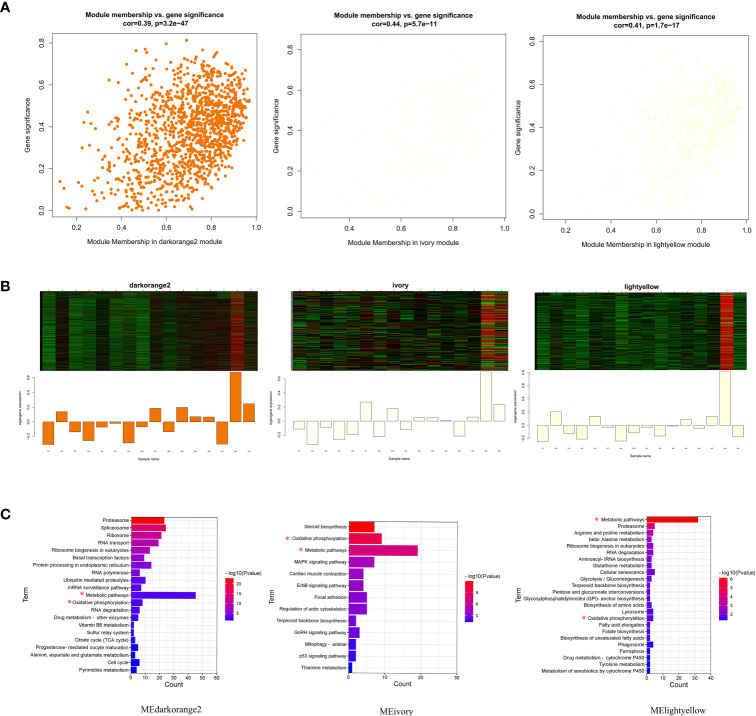
Visualization of gene significance (GS) vs. module membership (MM), expression levels and functional enrichment in three significantly related modules. **(A)** A scatterplot of GS for phagocytosis index and phagocytosis rate vs. MM in modules; **(B)** The heatmap and bar plot represent the expression level of genes in three modules. **(C)** Pathway enrichment analysis of all genes in three significantly correlated modules. Pathways coenriched by the three modules are marked with a red asterisk.

Functional enrichment of the genes in these significant modules revealed close linkages with phagocytosis. Three negatively correlated modules, MEdarkorange2, MEivory, and MElightyellow, together showed enrichment for metabolic pathways (most genes involved in this pathway) and oxidative phosphorylation (**Figures 5A–C** **;** **Supplementary Table S3**). Different modules were implicated in several different pathways: cellular senescence and phagosome for MElightyellow; MAPK signaling pathway and focal adhesion for MEivory; ubiquitin mediated proteolysis and cell cycle for MEdarkorange2.

**Figure 6:**
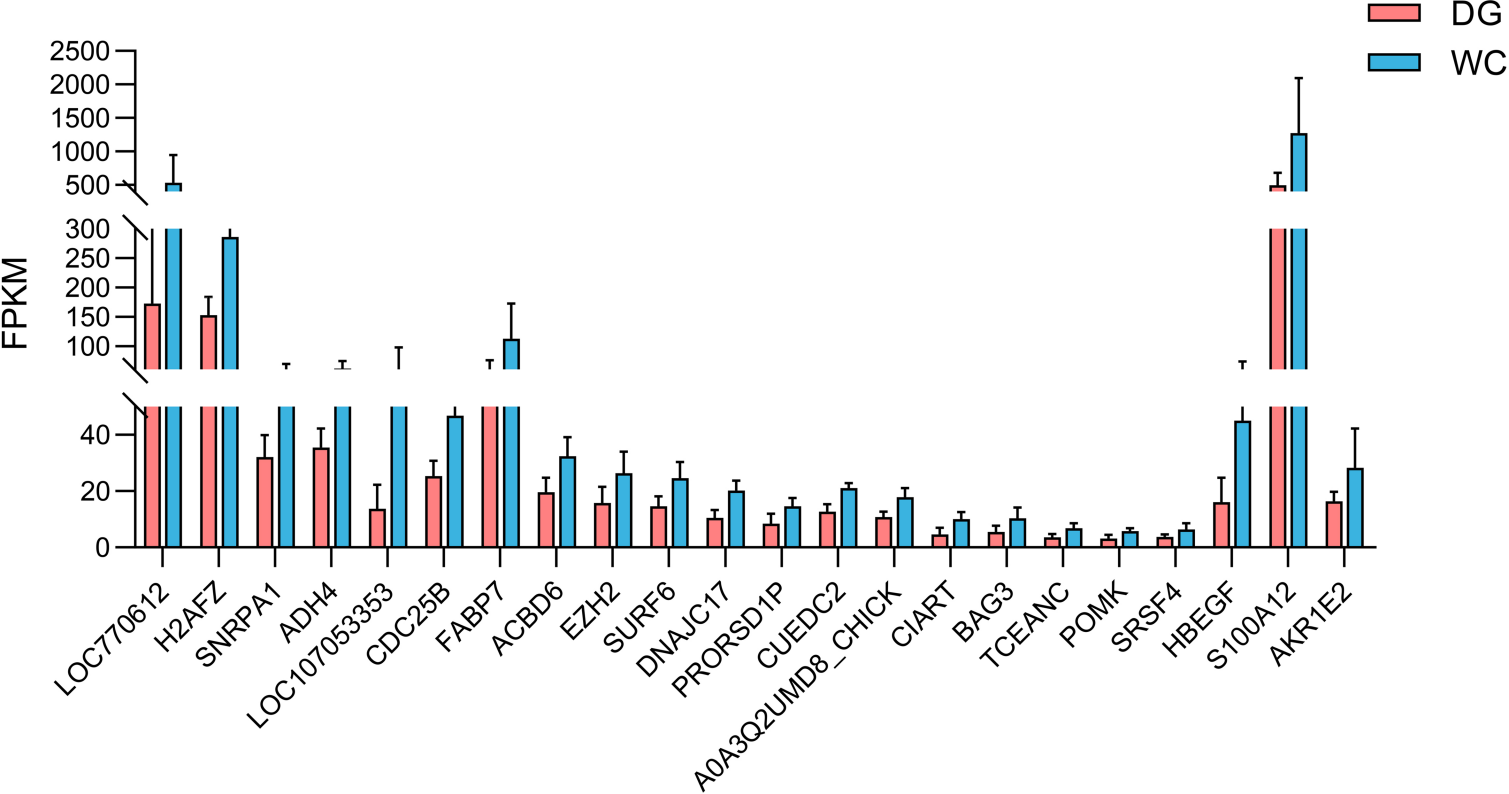
The expression levels of twenty-two intersecting genes of DEGs and hub genes in different modules. The default difference gene and the difference were not marked in the figure.

A total of 584 protein-coding genes were identified as hub genes in three significant modules (**Supplementary Table S4**). Furthermore, 2 MElightyellow hub genes, 1 MEivory hub gene and 19 MEdarkorange2 hub genes were differentially expressed. These genes were all significantly downregulated in Dagu chickens compared to Wenchang chickens (**Figure 6**). Notably, the MElightyellow hub gene *S100A12* is a member of the S100 family of proteins containing 2 EF-hand calcium-binding motifs involved in the innate immune response, including monocyte chemotaxis, neutrophil chemotaxis and positive regulation of NF-kappaB transcription factor activity (35). The MEdarkorange2 hub gene *CUEDC2* acts the negative regulation of macrophage cytokine production in the inflammatory response (36). These findings suggested that the mutual regulation of multiple genes and pathways affected the immune response of chicken macrophages.

### DEGs and hub genes were activated after stimulation by lipopolysaccharide in macrophage

**Figure 7:**
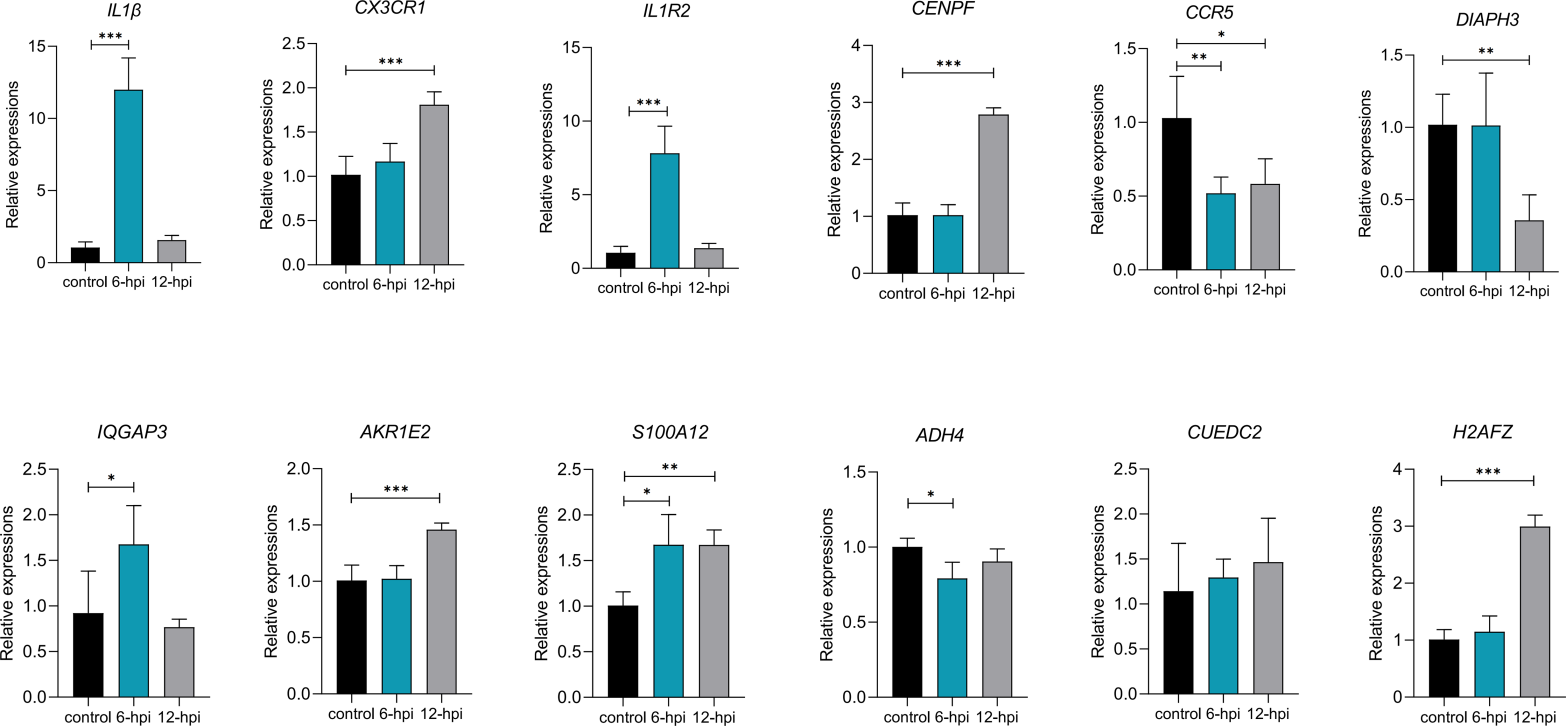
Some DEGs and hub genes mRNA expression after LPS stimulation in HD11 cells (n=3~6 per group). P < 0.05 (*), P < 0.01 (**) or P < 0.001 (***).

To verify whether the DEGs and hub genes screened in the above results were associated with inflammation, we detected the expression of these genes in macrophages after LPS stimulation in HD11 cells (**Figure 7**). The rapid increase in the expression of *IL1β* indicated that the construction of the inflammatory model was successful. The expression of *IL1R2* gene showed the similar trend as that of *IL1β* gene, which first increased and then decreased within 12 hours after LPS stimulation. Furthermore, *CX3CR1*, *CENPF*, *AKR1E2* and *H2AFZ* genes expressions increased significantly, while *CCR5* and *DIAPH3* genes expressions decreased significantly at 12 hours after LPS stimulation. Notably, the expressions of *CCR5*, *S100A12*, *IQGAP3* and *ADH4* genes differed significantly within 6 hours after LPS stimulation, indicating that these genes correspondingly antigen stimulated performed a faster immune response.

### *H2AFZ* gene under strong selection was intersection of DEGs and hub genes

**Figure 8:**
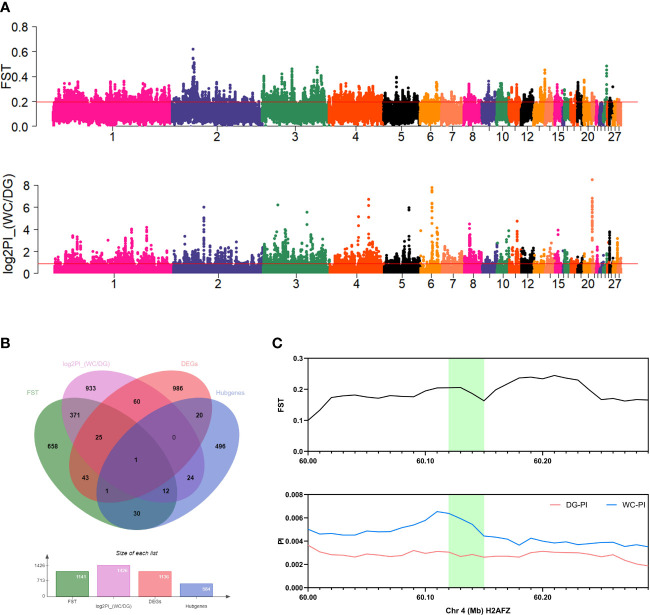
Population genomic analysis and gene colocalization. **(A)** Manhattan plots of FST and log_2_PI_(WC/DG) of 40 kb windows. Red lines indicate the top 5% threshold. **(B)** Venn diagram for the colocalization of FST, log_2_PI_(WC/DG), DEGs and hub genes. **(C)** FST value and PI value of colocalized genes in Dagu chickens and Wenchang chickens. Green fillers are the *H2AFZ* gene located in the two regions.

Comparative population genomics evaluated the population differentiation based on genomes of 30 Dagu chickens and 30 Wenchang chickens. A phylogenetic tree and PCA clearly showed population differences between the Dagu chickens and Wenchang chickens (**Supplementary Figure S4**). The top 5% windows based on the value of FST and log_2_PI_(WC/DG) were defined as strong repeatable signals of selection and visualized as manhattan plots (**Figure 8A**). In particular, 26 DEGs and 13 hub genes intersected with selected regions (**Figure 8B**). However, only the *H2AFZ* gene was identified among the DEGs and hub genes (**Figure 8C**). *H2AFZ* is a highly conserved gene encoding H2A.Z.1, mainly regulates cell cycle signaling and DNA replication (37).

## Discussion

Phagocytosis regulates the immune response and tissue homeostasis by ingesting and eliminating pathogens and apoptotic cells (34, 38). Many methods for measuring phagocytosis work on the principle of utilizing a variety of phagocytic targets with different combinations of receptor-ligand interactions. For example, pH-sensitive bioparticles or probes are efficient tools to study phagocytosis. Coupled with kinetic live cell imaging and automated analysis, the studies enable detailed quantitation of phagocytosis. In the current study, the phagocytosis of uniform fluorescent latex particles by peripheral blood macrophages was analyzed by flow cytometry (39). The percentage of phagocytosing macrophages (phagocytosis rate) and the number of internalized fluorescent latex particles per cell (phagocytosis index) were determined from cell size and fluorescence histograms.

Macrophages are effector cells of the innate immune system that recognize and remove bacteria, reflecting the strength of innate immunity. Many previous studies have reported differences in the level of the macrophage immune response across different chicken breeds. For example, chickens encoded by the *SAL1* locus on chromosome 5 exhibited more resistance to salmonellosis, and the resistance was expressed at the level of the mononuclear phagocyte system, such as the time to clear infected *Salmonella* and the respiratory burst response of macrophages. Since macrophage antimicrobial activity was associated with resistance, increased macrophage activity played a crucial role in the genetic resistance of chickens to systemic salmonellosis (40). In addition, Barbour EK et al. proved that the recruitment and phagocytic activity of peritoneal macrophages were significantly different among three different broiler breeds, which were closely related to resistance to *Salmonella enteritidis* (41). We assessed the phagocytic function of macrophages in Dagu chickens and Wenchang chickens, two local Chinese chicken breeds with obvious genetic differentiation. Dagu chickens exhibited the phagocytosis ability of peripheral blood macrophages, especially in the early stage of growth against *Salmonella* challenge. With increasing age, we found that the phagocytic ability of macrophages in Dagu chickens was still significantly higher than Wenchang chickens; however, the immune response level of the two chicken breeds to the same dose of *Salmonella* infection was not obvious. We hypothesized that 28 days of age is a turning point in the growth and development of chicks, and their immune function is relatively mature compared to the immune function of 14-day-old chicks, which better reflects the genetic characteristics of the breed.

The initiation and progression of macrophage phagocytosis are mediated by a complex, carefully orchestrated network of interactions and mediate the onset of other immune responses. To systematically evaluate the immune properties of the two breeds, other immune indicators were detected, including white blood cell count in peripheral blood and cellular immune factors in serum. The results showed that the percentage of macrophages and the H/L ratio in the peripheral blood of Dagu chickens were significantly increased than Wenchang chickens, especially in uninfected *Salmonella*. The H/L ratio was more sensitive to *Salmonella*, increasing rapidly 24 hours after *Salmonella* infection. In addition, the concentrations of IL17, IL23 and G-CSF in the serum of Dagu chickens were significantly lower than Wenchang chickens with or without *Salmonella* infection. We dissected these three cytokines as follows: interleukin 17 (IL17) is a proinflammatory cytokine, promoting M2 macrophage differentiation (42). Moreover, substantial evidence indicates that IL17 coordinates the accumulation of neutrophils in mammals, thereby contributing to host defense (43). IL17 also stimulates the phagocytosis of latex beads in mouse macrophages and human monocyte-derived macrophages. Interleukin 23 (IL23) promoted inflammatory outcomes in human macrophages, particularly pattern recognition-receptor (PRR)-induced intracellular bacterial clearance (44). Granulocyte colony stimulating factor (G-CSF), a key regulator of neutrophil production, is produced by activated monocytes and macrophages. Surprisingly, phagocytosis of apoptotic neutrophils by macrophages maintains approximately constant numbers in the peripheral blood *via* IL23, IL17 and the G-CSF-regulated axis (45). In this study, we speculated that the phagocytosis of macrophages and the number of peripheral blood leukocytes in Dagu chickens and Wenchang chickens are related to the concentration of the above three cytokines in serum, and the relationship among three cytokines, macrophage phagocytosis and peripheral blood leukocyte number requires more evidence.

Precise molecular genetic regulation is crucial for cell development and function. Transcriptomics and population genomics were combined to explore the genetic mechanisms underlying phagocytosis of macrophages in two Chinese native chicken breeds. DEGs between Dagu chickens and Wenchang chickens were assessed, and many biological pathways were identified that might be important to phagocytosis. Eighteen DEGs were mainly enriched in cytokine-cytokine receptor interaction pathways. A series of cytokines is essential for macrophages as sentinels of the innate immune system, mediating the transition from innate to adaptive immunity, and cytokine receptors can specifically bind to cytokines and transduce signal by cell surface glycoproteins (46). DEGs mentioned in the results, such as *CX3CR1*, *IL1R2* and *CCR5*, were involved in cytokine-cytokine receptor interaction pathways. *CX3CR1*, encoding a chemokine receptor, is known to regulate macrophage chemotaxis in injured tissue (47). *CCR5* encodes the chemokine receptor C-C motif chemokine receptor 5, which is expressed by T cells and macrophages and mediates macrophage chemotaxis (48). *IL1R2*, belonging to the interleukin 1 receptor family, was highly expressed in chicken macrophages in this study (acts upstream of or within the negative regulation of the interleukin-1-mediated signaling pathway). In mice, Il1r2(-/-) macrophages greatly enhances the production of inflammatory mediators in response to IL-1 (49). In addition, we also found that some DEGs were enriched mainly in the process of phagocytosis, such as regulation of actin cytoskeleton and phagosome pathways. Phagocytic processes is driven by the rearrangement of the actin cytoskeleton, and multiple signals converge at a phagosome to reorganize the actin cytoskeleton (50). *DIAPH3* and *IQGAP3* are involved in the regulation of actin cytoskeleton organization (51, 52). *DIAPH3* has been reported to be involved in actin remodeling, regulate cell movement and adhesion (51). *IQGAP3* has a function similar to that of *DIAPH3* gene and is involved in the regulation of actin cytoskeleton organization (52). Moreover, *IQGAP3* was reported to regulate intracellular signal transduction and macromolecule metabolic processes (53). Moreover, these genes were significantly different after LPS stimulation in macrophages.

Notably, phagocytosis is accompanied by metabolic changes, such as increased oxygen uptake. We screened hub genes of the three modules significantly related to phagocytosis that were collectively enriched in metabolic pathways and oxidative phosphorylation pathways. For example, *AKR1E2* and *POMK* were involved in metabolic pathways. *AKR1E2* (a member of the aldo-keto reductase superfamily) is characterized by its structure (evolutionarily highly conserved TIM barrel) and function (NAD(P)H-dependent oxido-reduction of carbonyl groups). NADPH is a cofactor used in reduction reactions, and seen in the anabolic pathways of organisms. *POMK* is involved in transmembrane junctions between extracellular matrix. POMK was predicted to regulate cytoskeleton and the processing of glycosylated proteins (54). Coincidentally, the expression levels of the two hub genes in macrophages of Dagu chickens were significantly lower than Wenchang chickens.

In addition, genome-wide selective sweep analysis revealed several genomic regions under selection between Dagu chickens and Wenchang chickens. These regions contain some genes, including *IL18*, *MAPK13*, *MAPK14*, *H2AFZ* and *PFN3*. Only *H2AFZ* was both a differentially expressed gene and a hub gene. We found that these selected genes were associated with immune responses, such as *Salmonella* infection, the MAPK signaling pathway, influenza A and the toll-like receptor signaling pathway. In particular, the strong selection on *H2AFZ* might be an adaptive response for robust immunity.

## Conclusions

Comparative functional analysis of macrophage phagocytosis revealed Dagu chickens performed stronger phagocytic ability than Wenchang chickens. Transcriptomic profile of macrophages revealed that DEGs and hub genes associated with phagocytosis, mainly participated different immunological biological signaling pathways. The target genes (e.g., *H2AFZ*) related to phagocytosis were identified by integrating transcriptomic and genomic analysis. The results facilitated a deeper understanding of the molecular mechanism of macrophage phagocytosis.

## Data availability statement

Transcriptome data of macrophages have been deposited in the Genome Sequence Archive repository (https://ngdc.cncb.ac.cn/gsa/), accession number CRA008360. The genome of Dagu chickens and Wenchang chickens are available from the corresponding author on reasonable request.

## Ethics statement

The animal study was reviewed and approved by all experimental procedures involving Dagu chickens and Wenchang chickens were performed according to the guidelines for experimental animals established by the Ministry of Science and Technology (Beijing, China). Ethical approval on animal survival was given by the animal welfare and ethics committee of the Institute of Animal Sciences (IAS), Chinese Academy of Agricultural Sciences (CAAS, Beijing, China) with the following reference number: IASCAAS-AE20140615.

## Author contributions

GZ, JZ and QW conceived the project and designed the research. JZ, QW, QL and ZW contributed to collecting samples and measuring phenotypic data. JZ and QW wrote the manuscript. GZ, MZ, JW revised the manuscript. All authors contributed to the article and approved the submitted version.
