# Supplementary material for: Comparative functional analysis of macrophage phagocytosis in Dagu chickens and Wenchang chickens
Source: Front Immunol. 2023 Feb 6;14:1064461. doi: 10.3389/fimmu.2023.1064461 (PMC9941738; doi:10.3389/fimmu.2023.1064461)
Supplement: Supplementary file 2 [file DataSheet_1.docx]

**Supplementary Figure 1**. Comparison of body weight between Dagu chickens and Wenchang chickens at 14-day-old and 28-day-old.

**Supplementary Figure 2**. Correlation analysis of phagocytosis index and phagocytosis rate.

**
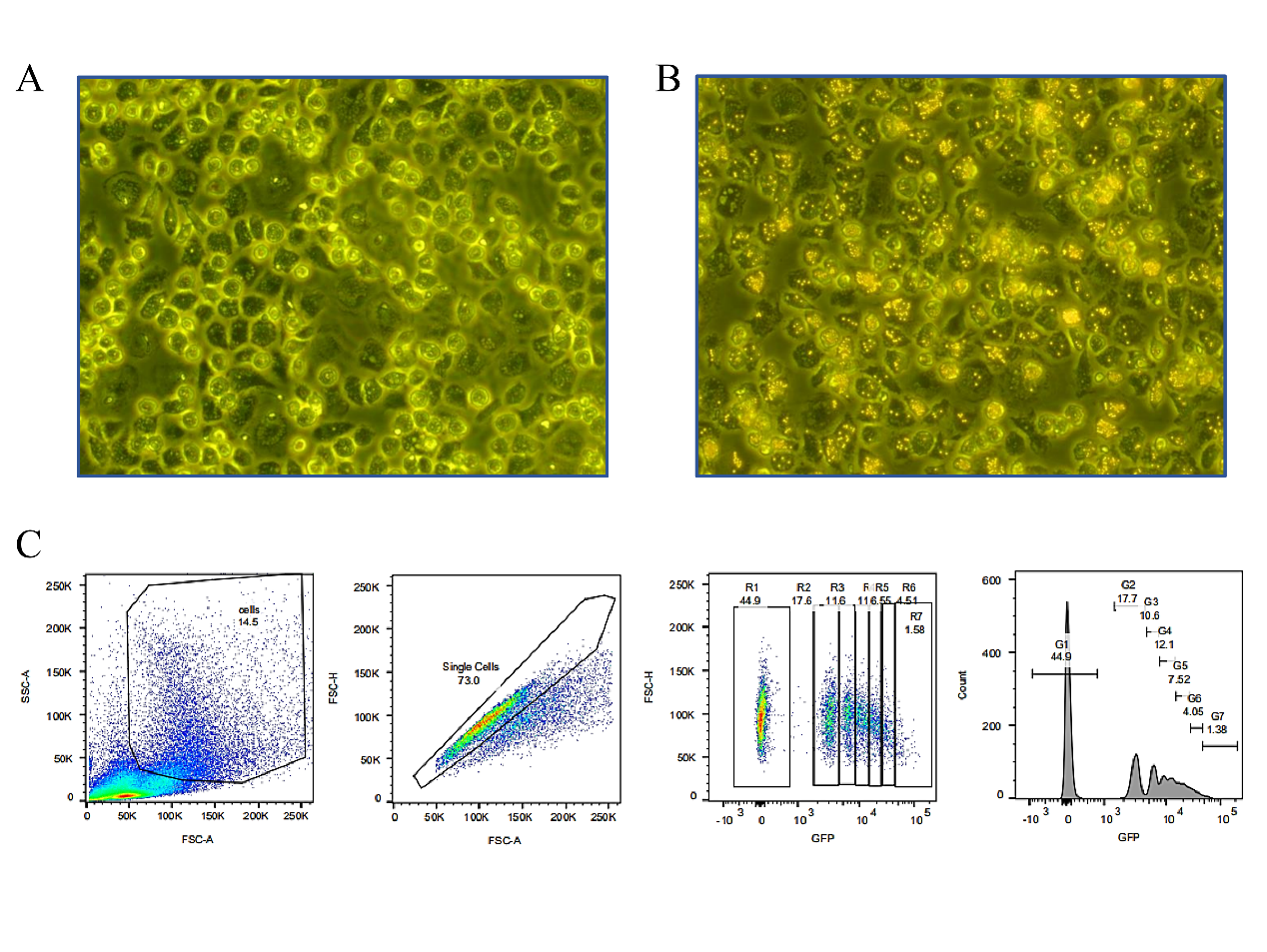
Supplementary Figure 3**. Macrophage phagocytosis assay. (A) Extraction and Culture primary Chicken macrophages (20×). (B) Macrophage phagocytosis of latex beads(20×). (C)The scatter plot of flow cytometry.


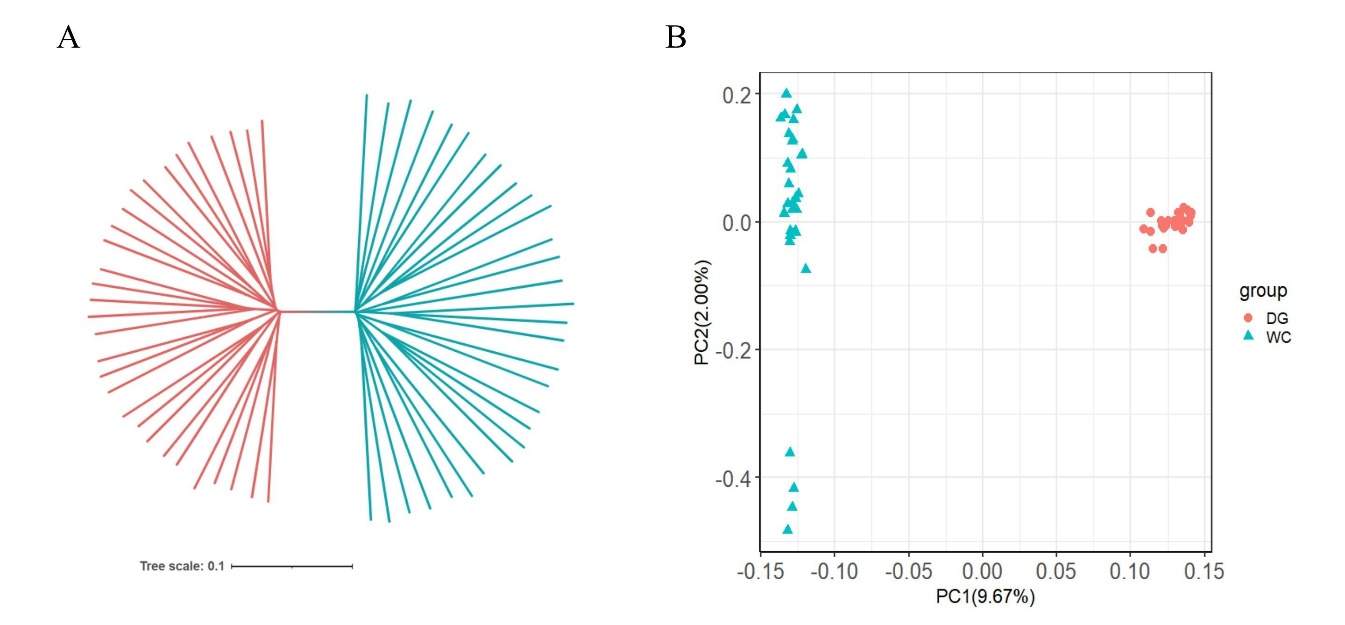


**Supplementary Figure 4**. Population genomic analysis. (A) Phylogenetic tree of Dagu chickens (red) and Wenchang chickens (blue). (B) PCA plot of all 60 individuals.
